# Primary breast tumours but not lung metastases induce protective anti-tumour immune responses after Treg-depletion

**DOI:** 10.1007/s00262-020-02603-x

**Published:** 2020-05-23

**Authors:** Ellyn Hughes, Sarah N. Lauder, Kathryn Smart, Anja Bloom, Jake Scott, Emma Jones, Michelle Somerville, Molly Browne, Andrew Blainey, Andrew Godkin, Ann Ager, Awen Gallimore

**Affiliations:** 1 Division of Infection and Immunity, Cardiff University School of Medicine, SIURI, Cardiff, CF14 4XN UK; 2 Cancer Research UK, Manchester Institute Cancer Biomarker Centre, University of Manchester, Alderley Park, Alderley Edge, Macclesfield, SK10 4TG UK

**Keywords:** T cells, Tregs, Resection, Breast cancer, Mouse, Immunotherapy

## Abstract

Although metastatic disease is responsible for the majority of cancer deaths, tests of novel immunotherapies in mouse tumour models often focus on primary tumours without determining whether these therapies also target metastatic disease. This study examined the impact of depleting Foxp3^+^ regulatory T cells (Treg), on lung metastases, using a mouse model of breast cancer. After Treg-depletion, generation of an immune response to the primary tumour was a critical determinant for limiting development of metastasis. Indeed, resection of the primary tumour abrogated any effect of Treg-depletion on metastases. In addition, whilst the immune response, generated by the primary tumour, prevented metastases development, it had little impact on controlling established disease. Collectively, the data indicate that metastatic cells in the lung are not controlled by immune responses induced by the primary tumour. These findings indicate that targeting Tregs alone will not suffice for treating lung metastases.

**Electronic supplementary material:**

The online version of this article (10.1007/s00262-020-02603-x) contains supplementary material, which is available to authorised users.

## Introduction

Metastasis is the primary cause of morbidity and mortality for individuals with cancer, accounting for around 90% of patient deaths [1–3]. In order for metastasis to occur, disseminated tumour cells must survive migration through the vascular or lymphatic system where they may become susceptible to immune attack, and extravasate into the new tissue, re-establishing tumours within a metastatic niche (reviewed in [2]). Given the bottlenecks involved, the process is inefficient, with only a few highly selected cells surviving the process. The immune system most likely represents a significant impediment to metastases since outside of the primary tumour site, circulating tumour cells may become vulnerable to immune attack; however, tumour-induced, systemic immunomodulation may aid their survival and dissemination to potential sites of metastases (reviewed in [4, 5]). Once a metastatic niche is established, locally acting immunosuppressive mechanisms may serve to promote growth of secondary lesions [6, 7].

CD4^+^Foxp3^+^ T cells (Treg) represent a potent arm of tumour-induced immunosuppression [8]. They are significantly enriched in many mouse and human tumours, and several studies have provided evidence that depletion of Treg promotes immune-mediated rejection at least of primary tumours ([9, 10] and reviewed in [8]). Few studies have examined the impact of Treg-depletion on spontaneous metastatic disease. This is a critical aspect of cancer immunotherapy as metastatic disease is the main cause of cancer-related death. In addition, it is known that Treg are often found at high frequencies in the circulation of cancer patients supporting the hypothesis that their presence aids survival of disseminated tumour cells (reviewed in [11]). To address this, we have used the 4T1 mouse breast cancer model. Following inoculation of 4T1 cells and subsequent growth in the mammary fat pad, these tumours spontaneously metastasise first to the lung, followed by liver, brain and bone. The tumour is poorly immunogenic and triple negative for common breast cancer markers [12–14]. With these characteristics in mind, 4T1 tumours have been widely used as a clinically relevant model for studying different stages of breast cancer progression and for testing drugs designed to impede malignancy (reviewed in [15]). Furthermore, as primary 4T1 tumours can easily be surgically excised, mimicking the treatment of human breast cancer [12–14], the impact of therapy on metastatic disease can be assessed in the presence or absence of the primary tumour. Specifically, we used this model to address the impact of Treg on breast cancer metastasis. We tested whether any effect of Treg-depletion on metastatic disease was linked to primary tumour size and/or the ability to mount an efficient immune response to the primary tumour. In addition, we examined whether there was any impact of Treg-depletion on establishment or progression of metastatic disease.

## Materials and methods

### Mice

DEpletion of REGulatory T cell (DEREG) mice, developed by Professor Tim Sparwasser, are a strain of BALB/C mice developed using a bacterial artificial chromosome (BAC) containing the *foxp3* locus and a diphtheria toxin receptor (DTR)-eGFP fusion protein inserted into the first exon of the *foxp3* gene [16]. As described previously, the Treg of DEREG mice can be selectively ablated by injection of diphtheria toxin (DTx). DEREG mice and littermates were bred and housed in filter-top cages in specific pathogen-free condition. Experiments were performed in accordance with Home Office UK guidelines.

### Analysis of Treg by flow cytometry

Single-cell suspensions prepared from tumours, spleens and LNs were subjected to red blood cell lysis buffer and subsequently stained using a fixable dead cell staining kit (LIVE/DEAD Aqua, Invitrogen). Cells were then surface stained with CD4-specific antibodies (Biolegend) before fixation/permeabilisation (eBioscience) and staining with Foxp3-specific antibodies (eBioscience). Fixed cells were run through a Novocyte (ACEA) flow cytometer and analysed using FlowJo software.

### Culture and injection of 4T1 cells

4T1 (obtained from ATCC (CRL-2539)), is a murine mammary carcinoma that closely mimics stage 4 triple-negative human breast cancer. The cells were maintained in complete RPMI medium supplemented with 10% Foetal Calf Serum (FCS) 2 mM L-glutamine, 1 mM sodium pyruvate, 50 μg/ml penicillin and streptomycin at 37 °C, 5% CO_2_. Cells were split up to 3 times a week and grown for 3–6 passages before being injected into the mammary fat pads of DEREG mice. Tumours were measured up to 3 times per week from day 7 until the tumour was resected or until the mouse was killed. Two perpendicular measurements were taken of the tumour from which the volume was calculated. Tumour volume = (length × width × short)*(3.14/6), [where short equals the lower of the length and width measurements and provides an estimate of height] [10]. In some experiments 4T1 cells were injected intravenously into the tail vein.

### Surgical removal of primary tumour

Tumours were measured before induction of anaesthesia (isofluorane) and subcutaneous injection with the anti-inflammatory analgesic, Metacam (Boehringer Ingelheim). The tumour and the surrounding area were shaved and swabbed with Hibiscrub antibacterial wash and surgical spirit. Aseptic techniques were used to excise the tumour and close the wound using horizontal mattress sutures (Ethicon). Mice, placed in a heated recovery chamber until consciousness was regained, were monitored postoperative for well-being and suture strength.

### Cancer vaccination

For primary tumour control studies prior to tumour inoculation 1 × 10^7^ plaque-forming units (pfu) of vaccinia virus expressing the 4T1 immunodominant peptide epitope, SPSYVYHQF, named AH1 (Vac-AH1) or vaccinia expressing influenza nucleoprotein (Vac-NP) as an irrelevant control, were administered i.p [9, 17, 18]. Fourteen days post-vaccination the antigen-specific response was boosted by a second vaccination with either 1 × 10^7^ pfu of Vac-AH1 or Vac-NP. 4T1 tumours were inoculated 7 days later. For studies relating to metastatic control, following resection of primary tumours, mice were administered DTx or PBS as indicated every other day from day 1 postsurgery. Mice were vaccinated at day 2 postresection with either 1 × 10^7^ pfu of Vac-AH1 or Vac-NP. To determine vaccination efficacy, splenocytes were stained 14 days postvaccination using a dead cell staining kit (LIVE/DEAD Aqua, Invitrogen). Cells were stained with a H-2L^d^ PE-labelled dextramer comprising peptide SPSYVYHQF (Immudex) and subsequently surface stained with CD3-, CD4- and CD8-specific antibodies. Cells were run through a Novocyte (ACEA) flow cytometer and analysed using FlowJo software.

### Analysis of CD8^+^ IFN-γ production by flow cytometry

Intracellular IFN-γ staining of CD8 T cells was performed using single-cell suspensions derived from lungs. Cultures were stimulated at 37 °C in complete RPMI with phorbol12-myristate 13-acetate (PMA, Sigma-Aldrich) (20 nM) and ionomycin (1 μg/ml) (Sigma-Aldrich) for 5 h; brefeldin (1 μg/ml, Biolegend) was added for the last 4 h. After incubation cells were stained using a fixable dead cell staining kit (LIVE/DEAD Aqua, Invitrogen). Cells were then surface stained with CD8-specific antibodies (Biolegend) before fixation/permeabilisation (BD Biosciences) and staining with IFN-γ-specific antibodies (Biolegend). Fixed cells were run through a Novocyte (ACEA) flow cytometer and analysed using FlowJo software.

### Clonogenic assay

Lungs were mechanically disrupted prior to digestion in collagenase IV cocktail (Sigma-Aldrich) at 4 °C for 75 min. Each sample was subsequently passed through a 70-μm nylon cell strainer before resuspension in 10 ml of 6-TG media (60 mM 6-thioguanine (Sigma-Aldrich)) in 1 M sodium hydroxide solution diluted to 60 μm 6-thioguanine in 10% IMDM (IMDM supplemented with 10% FCS, 2 mM L-glutamine, 1 mM sodium pyruvate, 50 μg/ml penicillin and streptomycin). Lung suspensions were plated onto 9-cm tissue culture plate (Greiner) and incubated for 14 days at 37 °C, 5% CO_2_. After 14 days in culture, media were discarded, and the plates were fixed with 5 ml of methanol for 5 min. Plates were washed with sterile water and stained with 0.03% methylene blue dye (Sigma-Aldrich) for 5 min prior to washing twice with sterile water. Plates were left to dry, and the resulting blue colonies were counted, with each blue colony representing one metastatic cell [14].

### In Vivo* Treg depletion*

DTx (native antigen) was injected intraperitoneally (i.p) at a concentration of 5–15 μg/kg every other day. For assessing the effects of Treg depletion on the primary tumour, DTx was injected i.p from the day the primary tumour was palpable (day 5). For assessing the effects of Treg depletion on metastasis, mice were injected with DTx twice before surgery on day 0 (day 4 and day 2) or every two days after surgery until mice were killed. Control mice were either DEREG littermate controls injected with DTx as above or Treg-replete mice were injected i.p. with PBS at the stated time points.

### Lung immunohistochemistry

Lungs were perfused with 5 ml of PBS and fixed in 10% neutral-buffered formalin in saline. Lungs were embedded into paraffin, and 5-μm sections were cut. To determine metastatic burden lung sections were stained with haematoxylin and eosin and then scanned using a Zeiss Axio Scan.Z1 to visualise the entire lung. To enumerate CD3^+^ T cell infiltration into the metastatic nodules, lungs were stained with anti-CD3 antibodies (Dako) prior to detection with DAB chromogen and counterstaining with haematoxylin. Slides were scanned and CD3^+^ T cells enumerated per metastatic nodule using Zen software (Zeiss).

### Statistics

Statistical analysis was performed using Excel or GraphPad Prism 8. Unless stated otherwise in the figure legends, data are displayed as the mean ± SEM. Statistical significance is denoted as follows: *p < 0.05; **p < 0.01; ***p < 0.001.

## Results and discussion

### Treg depletion can promote control of metastatic disease

**Fig. 1:**
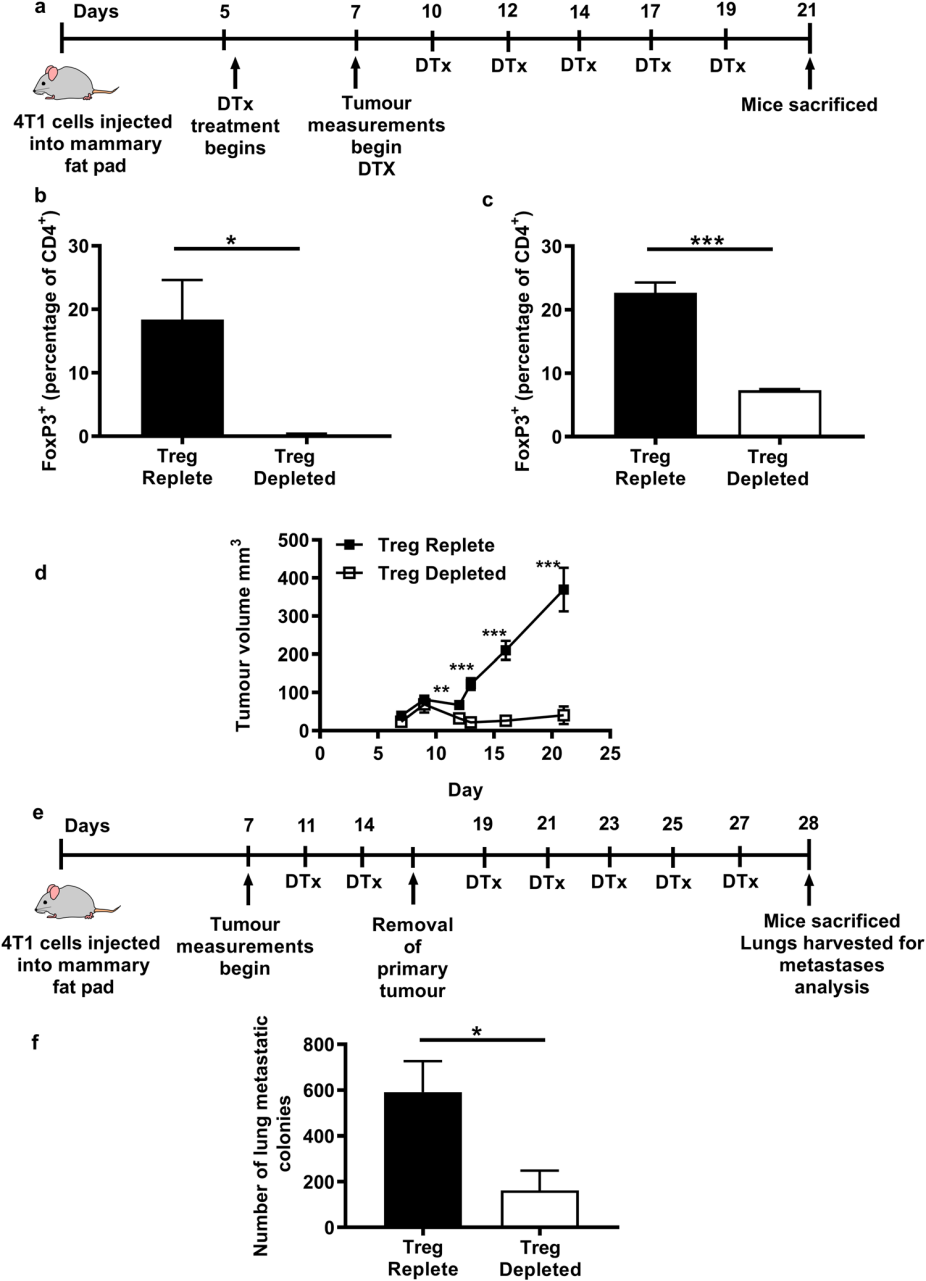
Depletion of Tregs can control growth of primary 4T1 tumours and metastatic disease. **a** The effect of Treg-depletion on primary tumour growth was assessed as outlined. **b** The number of Tregs present within tumours or **c** tumour draining lymph nodes 7 days after administration of DTX (4 mice/group). An unpaired t test was performed to determine significance (*P* = 0.0268). **d** The average volumes of 4T1 tumours in Treg-depleted and Treg-replete mice are shown (6–7 mice/group). An unpaired two-tailed t test was performed at each time point, giving the following values: day 12 *P* = 0.0034, day 13 *P* =  < 0.0001, day 16 *p* =  < 0.0001 and day 21 *p* = 0.0002. **e** The impact of Treg-depletion on metastases following resection of primary tumours was assessed as outlined. **f** The number of metastatic colonies observed in the lungs of Treg-depleted and Treg-replete mice is shown (17–18 mice/group). A Mann–Whitney test was performed to determine whether there was a statistically significant difference between the groups (*p* = 0.0162)

The potential of immunotherapy to cure metastatic disease is demonstrated by the success of treating certain cancers, most notably metastatic melanoma, but it remains the case that the vast majority of cancers still do not respond to these treatments. This may reflect a lack of knowledge of the relationship between the immune system and metastases. In the case of preclinical mouse models, novel therapies are typically tested on primary tumours and not on metastatic disease. For this reason, we examined the effect of Treg depletion in a clinically relevant mouse model of breast cancer. We hypothesised that as primary tumour control was promoted through Treg depletion, metastases would also be controlled in the same animals. In order to generate primary tumours, DEREG-positive mice and their negative littermates were injected subcutaneously with 4T1 cells with tumours becoming palpable around 5 days later. At this point, injections with DTx were started and continued every other day until killing on day 21. Injections of DTx successfully depleted Tregs in tumours and lymph nodes (Fig. 1b and c). Tumours in Treg-replete mice grew uniformly, whereas many tumours in Treg-depleted mice began to regress after day 10, with some tumours demonstrating complete regression (Fig. 1d).

To determine whether DTx treatment impinged on metastases, mice were given two rounds of treatment with DTx on days 10 and 12 before primary tumours were resected on day 14. A comparison of metastatic colonies revealed significantly fewer colonies in Treg-depleted mice (Fig. 1e and f) and significantly fewer Treg-depleted mice with any metastatic colonies compared to Treg-replete mice (75 versus 25%), indicating that Tregs do indeed promote disease progression in this model, since their depletion limits the number of mice with metastatic nodules in the lung.

### Control of primary tumour growth predicts control of metastatic disease following Treg-depletion

**Fig. 2:**
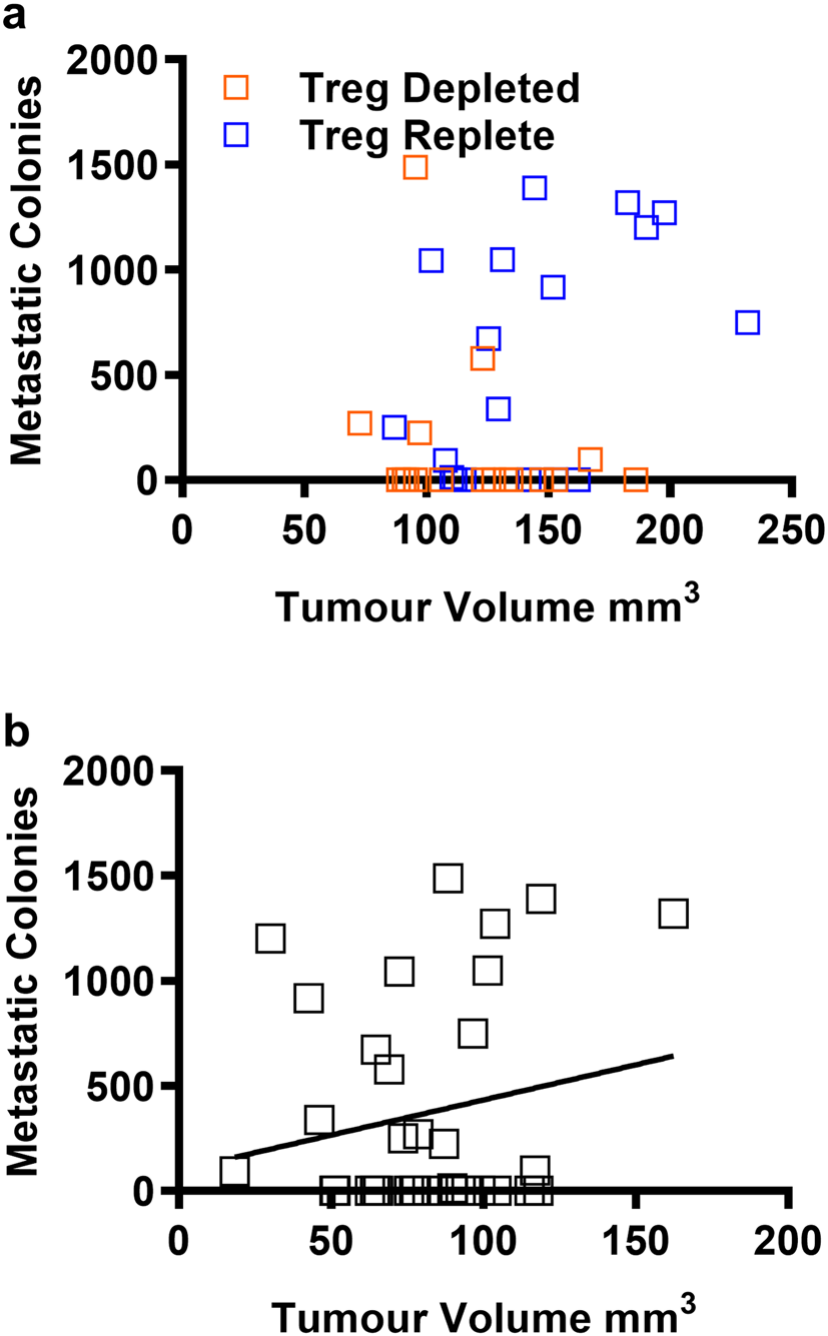
Control of metastatic disease after Treg-depletion is not linked to primary tumour size. The number of metastatic nodules was compared to the size of the primary tumour at the point of resection **a** or **b** the point at which DTx treatment was initiated (17–18 mice/group)

**Fig. 3:**
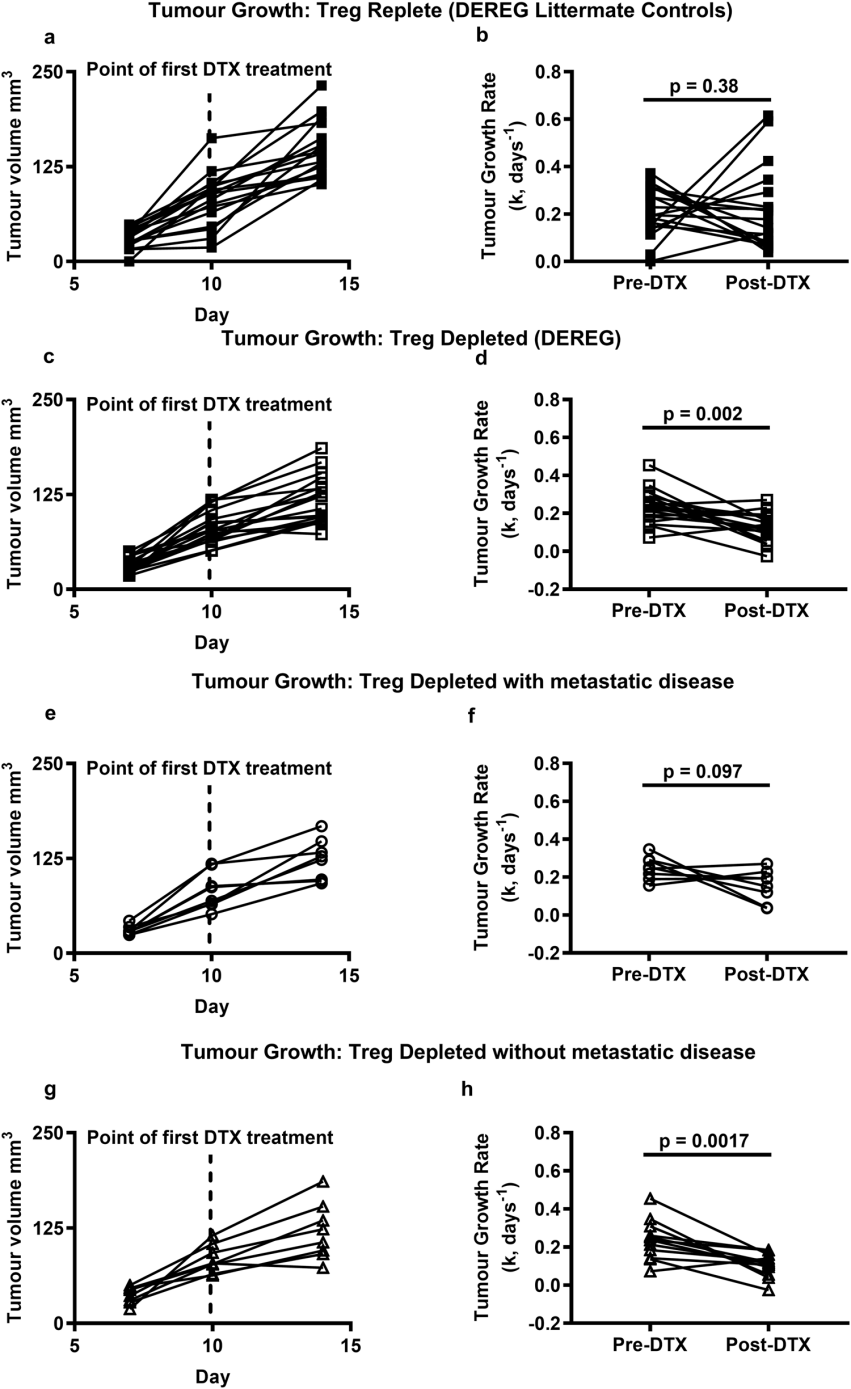
Control of primary tumour growth predicts control of metastatic disease following Treg-depletion. Tumour growth rates were assessed in Treg-replete (**a**) and Treg-depleted (**c**) mice and compared prior to and after administration of DTx (**b**, **d**) (17–18 mice/group). Within the Treg-depleted group, mice were split into those with metastatic disease (**e**, **f**) and those without (**g**, **h**) (8–12 mice/group). A nonparametric one-tailed Wilcoxon signed-rank test was performed on the paired data sets shown in individual graphs

Since metastatic disease was not reduced in all mice after Treg-depletion, we next examined whether control of metastatic disease, observed in some Treg-depleted mice, was linked to the size of the primary tumour at the point of Treg-depletion or primary tumour resection. No significant associations were observed between numbers of metastatic nodules and the size of the primary tumour either at the point of Treg-depletion or at the point of resection indicating that primary tumour size does not predict control of metastasis after Treg-depletion (Fig. 2a and b). Primary tumour growth rates were measured before and after DTx treatment prior to tumour resection and compared to tumour growth rates over the same time course in Treg-replete mice (DTx-treated DEREG-negative littermates). As expected, in the Treg-replete group, tumour growth rates remained unchanged overall at the different time points (Fig. 3a and b), whereas overall, tumour growth rates decreased in the Treg-depleted group (Fig. 3c and d). We next split the Treg-depleted group into those which developed metastases (Fig. 3e and f) and those which did not (Fig. 3g and h) and examined whether there was an association with individual tumour growth rates measured before and after Treg depletion. The data clearly indicate that the presence of metastases is associated with a failure to control the primary tumour after Treg-depletion. These findings are in line with a study performed by Liu and colleagues [19]. Whilst metastatic load was not measured as part of their study, they showed that the overall survival of tumour-bearing mice depleted of Tregs or treated with anti-PD1/anti-CD137 antibodies was significantly longer when the immunotherapy was administered prior to rather than after tumour resection [19]. This was associated with development of a CD8^+^ T cell response to the L^d^-restricted peptide epitope, AH1 [17]. Both studies point to a critical requirement for the primary tumour to drive development of an immune response after either Treg-depletion or co-inhibitory receptor blockade [19].

These findings are interesting in the context of previous reports indicating that primary 4T1 tumours cause generalised immunosuppression that is reversed following surgical resection; a finding that implies that tumour resection should aid induction of an anti-tumour immune response and not compromise it [12]. These studies however specifically compared immune responses to foreign or allogeneic antigens in the presence and absence of the primary tumour, revealing that both antibody and T cell responses generated against these antigens were significantly improved after tumour removal [12]. Our observations imply that Tregs play a part in tumour-driven immunosuppression but also show that after Treg depletion, the continued presence of the primary tumour is critical for enabling development of a successful anti-tumour T cell response. Thus, whilst the primary tumour does cause immunosuppression, this tumour is an important source of tumour antigens needed to drive the anti-tumour response when the immunosuppression is reversed. These findings are relevant since currently most breast cancer patients undergo surgery before other therapies such as chemotherapy, radiation therapy, hormone therapy and possibly immunotherapy. There is however a growing body of evidence from preclinical and clinical studies of the potential benefit of neoadjuvant immunotherapy [19, 20]; our data support this concept clearly showing that surgical removal of a solid primary cancer has a negative impact on the development of an anti-tumour response after manipulating Treg.

### Treg-depletion does not limit metastatic disease after resection of the primary tumour

**Fig. 4:**
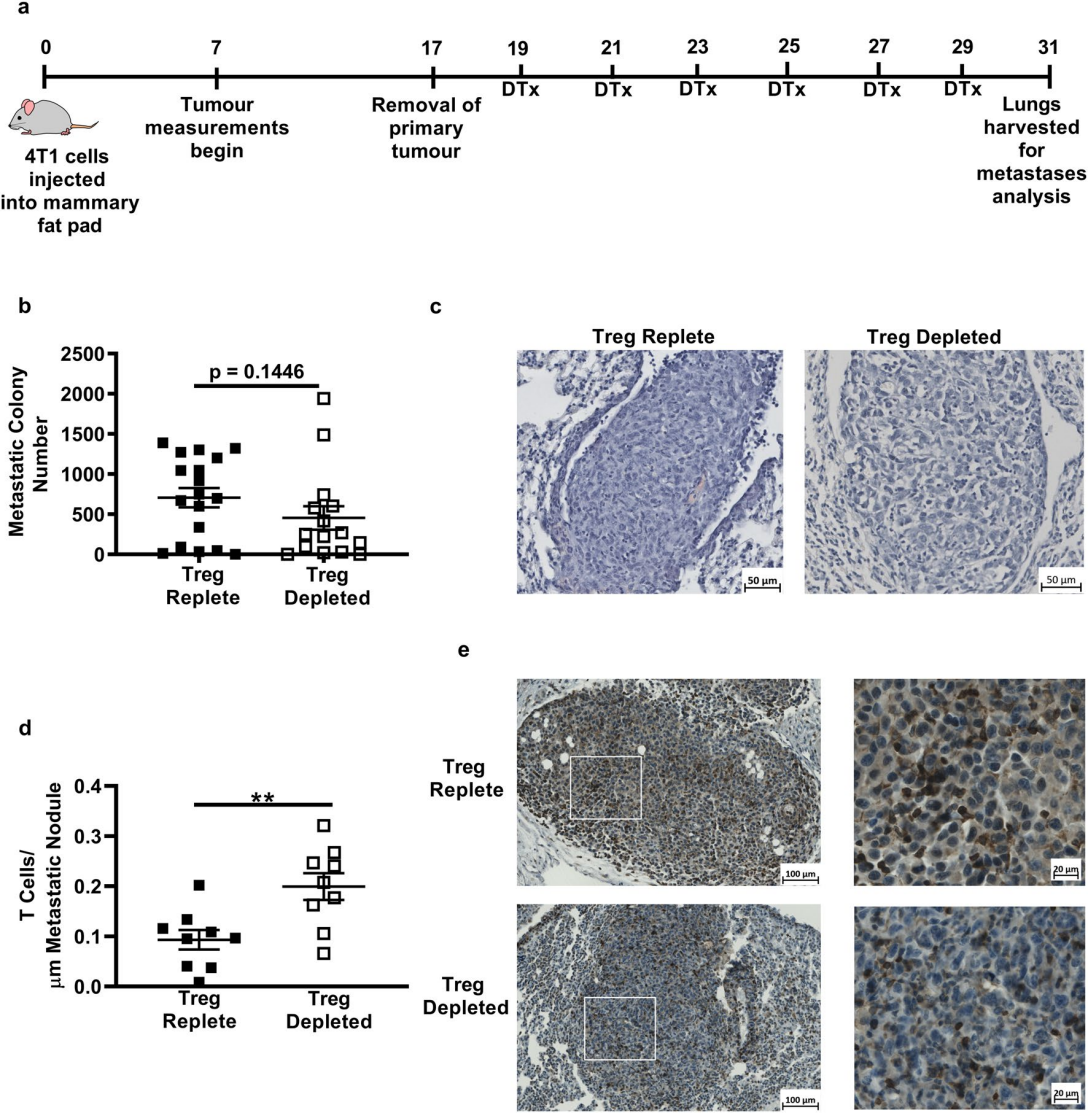
The presence of the primary tumour is essential for promoting control of metastatic disease after Treg-depletion. **a** DTx treatment was started after 4T1 tumours were resected. **b** Metastatic colonies in Treg-depleted and Treg-replete mice were compared, a Mann–Whitney test was used to determine no significant difference was observed between the number of metastatic colonies in Treg-replete versus Treg-depleted mice (15–18 mice/group). **c** Haematoxylin and eosin staining of paraffin-embedded sections from Treg-replete versus Treg-depleted metastatic lungs. **d** The number of CD3^+^ T cells (brown) per μm of metastatic nodules was calculated in the lungs of Treg-replete and Treg-depleted mice (9 metastatic nodules/group). **e** Representative staining of CD3^+^ T cells in metastatic lung nodules from Treg-replete and Treg-depleted mice

Since control of the primary tumour following depletion of Treg was indicative of reduced metastatic disease, we sought to confirm that the primary tumour was essential for induction of the protective anti-tumour immune response. For this purpose, tumours were resected prior to administration of DTx in order to remove the primary tumour as a source of antigen for T cell priming following Treg depletion (Fig. 4a).

Histological analyses of tumour-bearing lungs show similar patterns of lymphocytic infiltrate and metastatic nodules of comparable size in Treg-replete and Treg-depleted mice (Fig. 4b and c). Staining and counting of T cells in metastatic nodules did however reveal a significantly higher number of tumour-infiltrating T cells in Treg-depleted mice (Fig. 4d and e). In spite of this, a comparison of metastatic colonies revealed no significant difference in the proportion of mice developing metastatic disease in Treg-depleted and Treg-replete groups (50% versus 64.2%), nor in the numbers of metastatic colonies observed in both groups (Fig. 4b).

Overall, these data show that metastatic tumours are not effectively controlled even after Treg-depletion revealing a fundamental difference in metastases versus primary tumours. This is compatible with data from a previous study which indicate that Treg-depletion does reduce the in situ formation of adenocarcinomas in a genetically engineered mouse model of nonsmall cell lung cancer (NSCLC) [21]. In this case, the inherent immunogenicity of primary tumours arising in the lung may enable induction of a productive immune response when Treg are absent, whilst metastatic tumour cells, which have already undergone immune editing, fall beneath a critical immunogenicity threshold.

**Fig. 5:**
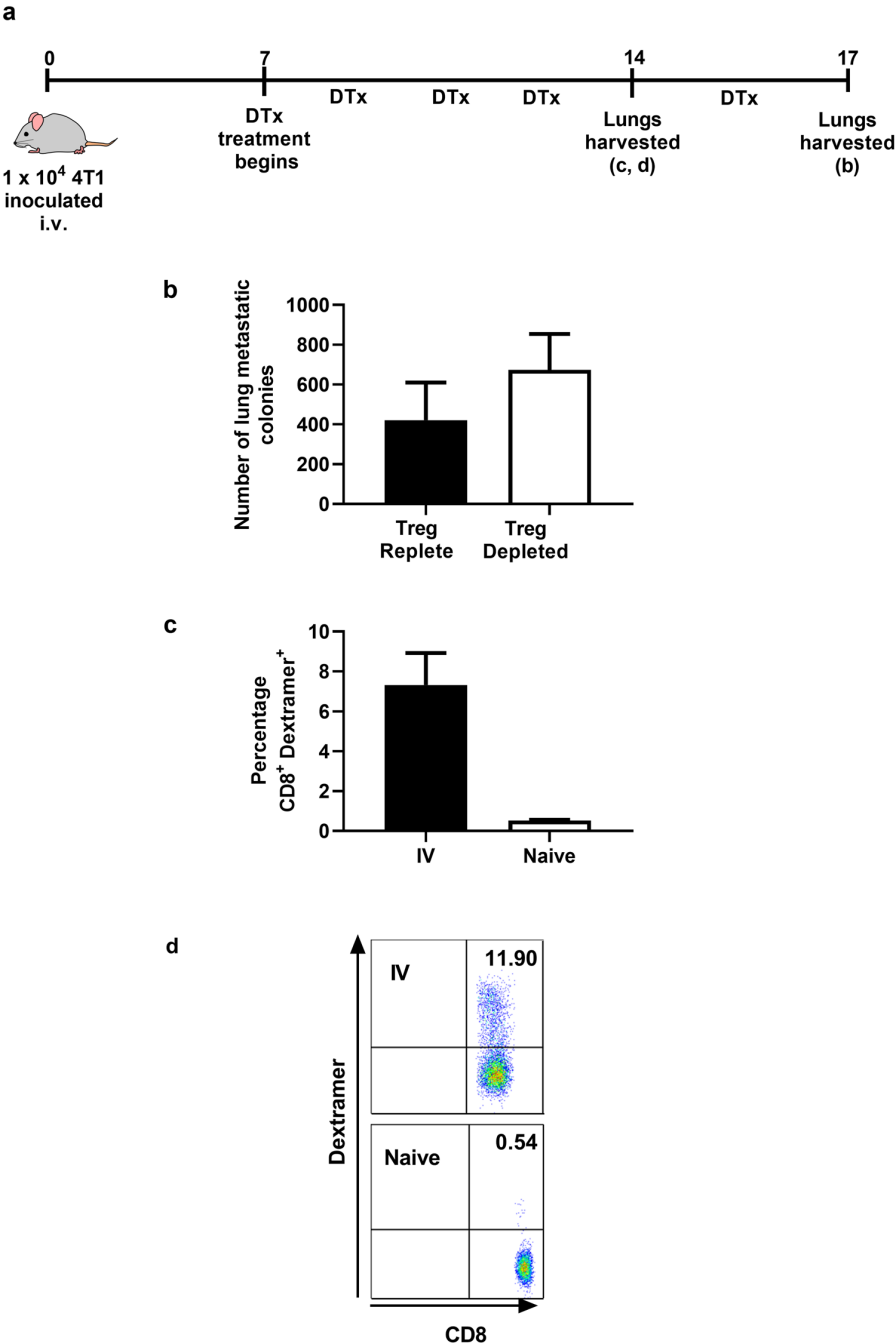
Treg-depletion does not result in control of 4T1 tumour growth after intravenous injection. **a** Mice injected intravenously with 10^4^ 4T1 cells and seven days later administered DTx or PBS every other day (6 mice per group). **b** Metastatic colonies were enumerated 17 days later. **c**, **d** Lung T cells were was stained with AH1-dextramers (3–6 mice/group). Statistical significance was determined by unpaired two tailed T test, *p ≤ 0.05

In the study described herein, where Treg depletion prior to resection of the primary tumours was shown to result in less metastatic disease, this is most likely due to limiting the establishment of metastatic nodules possibly through affecting dissemination, intravasation and/or survival of the tumour cells. The question arising from these data is why metastases in the lung do not induce productive immune responses after depletion of Treg, whilst the primary tumours growing in the mammary fat pad do? This cannot be explained by an inherent alteration in immunogenicity of the disseminated tumour cells, which eventually colonise the lungs compared to those in the primary tumour as even after delivery of a primary inoculum of 4T1 cells via the i.v. route, we see no difference in numbers of lung colonies forming in Treg-replete and Treg-depleted mice (Fig. 5b). This finding cannot be explained by a complete inability of i.v.-injected tumour cells to induce T cell responses since antigen-specific T cells, measured using dextramers comprising the AH1 peptide [17] can be seen in the lungs of i.v.-injected mice (Fig. 5c and d). Overall, these data imply that failure to control metastatic nodules in the lung is not due to a failure to induce T cells or a failure of T cells to infiltrate the lung.

**Fig. 6:**
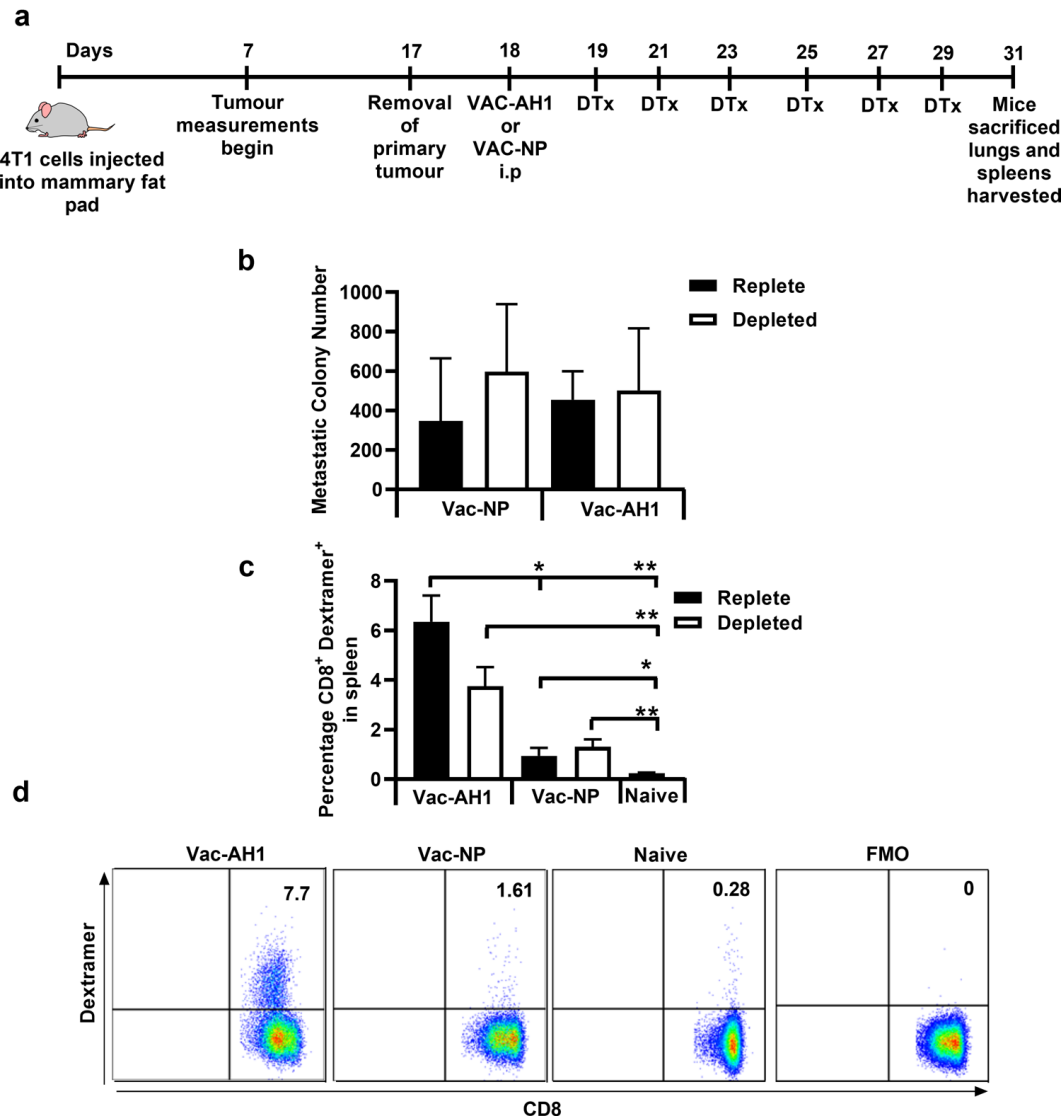
Immunisation following tumour resection, with recombinant vaccinia virus expressing the immunodominant peptide AH1, promotes tumour-specific T cells but does not control metastatic disease. **a** Mice were either treated with an irrelevant vaccine (Vac-NP) or a tumour antigen-specific vaccine (Vac-AH1) at day 1 post tumour resection and then either DTx or PBS treated from day 2 post tumour-resection,. At day 14 post tumour resection **b** the metastatic colonies in Treg-replete and Treg-depleted mice were compared (3—10 mice/group) and **c** splenocytes were stained with AH1-dextramer (3—10mice/group). **d** Representative CD8^+^ AH1-dextramer^+^ flow cytometry plots from mice (shown in **c**) vaccinated with either tumour-specific VAC-AH1, irrelevant VAC-NP or tumour-naïve mice

We sought to confirm this finding in the case where 4T1 cells colonise the lungs naturally as a result of dissemination from the primary tumour. For this purpose, we tested whether vaccination with the immunodominant 4T1 antigen [17], AH1, led to improved control of metastases. Thus, mice were treated as shown in Fig. 4a, Treg-depleted but in addition, administered recombinant vaccinia virus expressing AH1 (Vac-AH1 [9]) or a control virus expressing influenza nucleoprotein (Vac-NP [18]) at day 1 post resection. Vac-AH1 was used as it 1. expresses the immunodominant 4T1 L^d^-restricted peptide SPSYVYHQF (AH1), 2. elicits AH1-specific T cells (Supplementary Fig. 1a, b and c) and 3. results in a significant control of 4T1 tumours in immunised mice (Supplementary Fig. 1d). Moreover, this experiment clearly demonstrated that CD8^+^ T cells in the lungs of tumour-bearing mice are functional as assessed by their ability to produce IFN-γ (Supplementary Fig. 1e and f). Despite the ability of Vac-AH1 to induce T cell capable of both controlling the primary tumour and infiltrating the lung, immunisation after resection of the primary tumour had no impact on control of metastatic disease, despite clearly eliciting an AH1-specific T cell response (Fig. 6a–d).

Overall, our data indicate that once established, metastases progress regardless of whether Treg are depleted and resist attack from the immune response generated by the primary tumour. This is the case even when antigen-specific T cell responses are clearly present in the lung. Whilst this may be due in part to immune-editing mechanisms, local barriers to immune attack are likely to be multi-factorial, resulting from the concerted actions of many cell types including platelets, immunosuppressive B cells and T cells, monocytes and macrophages [22–24]. The latter may be fundamental in the development of pulmonary metastases, as Qian et al. demonstrated that the CCL2-mediated recruitment of inflammatory monocytes and subsequently metastasis-associated macrophages (MAMs) occurs prior to infiltration of Treg. Furthermore, disruption of CCL2-CCR2 signalling targeted the MAMs and prevented metastatic seeding, with limited effect upon Treg [25]. CCL2 recruits inflammatory monocytes to facilitate breast tumour metastasis [25]. This study highlights differences in the immune composition of primary tumours and metastases; this distinction may reflect trafficking mechanisms which dictate the direction and extent to which immune cells access different organs.

In the case of 4T1 cells, there is evidence that myeloid-derived suppressor cells (MDSC) create a premetastatic niche through production of the exosomal protein S100A8/A9 [26], which may serve to protect subsequent tumour growth from immune attack, even when Treg are absent. There is widespread interest in the role of exosomes derived from malignant cells in premetastatic niche formation. Costa-Silva demonstrated in a model of pancreatic cancer that uptake of tumour-associated exosomes by the kupffer cells of the liver resulted in the development of a fibrotic environment [27]. This favoured the recruitment of MDSC and macrophages and the formation of a premetastatic niche within the liver. Subsequent studies focussing on the development of lung premetastatic niches have shown a similar effect but with a preference for the recruitment of neutrophils [28]. As reviewed by Peinado et al. it is known that different tumours and their associated exosomes will form a premetastatic niche in organs that are conducive to their specific growth needs [29]. It is possible that the exosome-induced development of metastases in the lungs of 4T1 mice is a process independent of Treg control.

An accumulating body of evidence also indicates that particular subsets of cancer-associated fibroblasts (CAF) are a major immunosuppressive influence in many cancers, including cancers of the breast ([30] and reviewed in [31]). By a combination of single-cell RNA sequencing and multi-parameter phenotypic analyses, it is evident that CAF are highly heterogeneous, comprising cell populations of different origins and function. Recently, Costa and colleagues showed that a particular subset of CAF drives immunosuppression in triple-negative breast cancer [30].

Studies performed in both mice and human suggest that several barriers must be overcome to enable induction of effective anti-metastatic immune responses in the postresection (adjuvant) setting [32–34]. Our data suggest that inclusion of a cancer vaccine may still require additional immune-modulating regime(s), possibly based on targeting MDSC or distinct CAF subsets. Other possibilities include concomitant delivery of chemotherapy (to induce local immunogenic cell death) or targeted radiotherapy [35]. These are considerations worth exploring since delay in surgical excisions may not always be possible or to the overall benefit of the patient.

### Electronic supplementary material

Below is the link to the electronic supplementary material.

Supplementary file1 (TIF 2810 kb)
