# Supplementary figures and images for: Primary breast tumours but not lung metastases induce protective anti-tumour immune responses after Treg-depletion
Source: Cancer Immunol Immunother. 2020 May 23;69(10):2063–73. doi: 10.1007/s00262-020-02603-x (PMC7511476; doi:10.1007/s00262-020-02603-x)

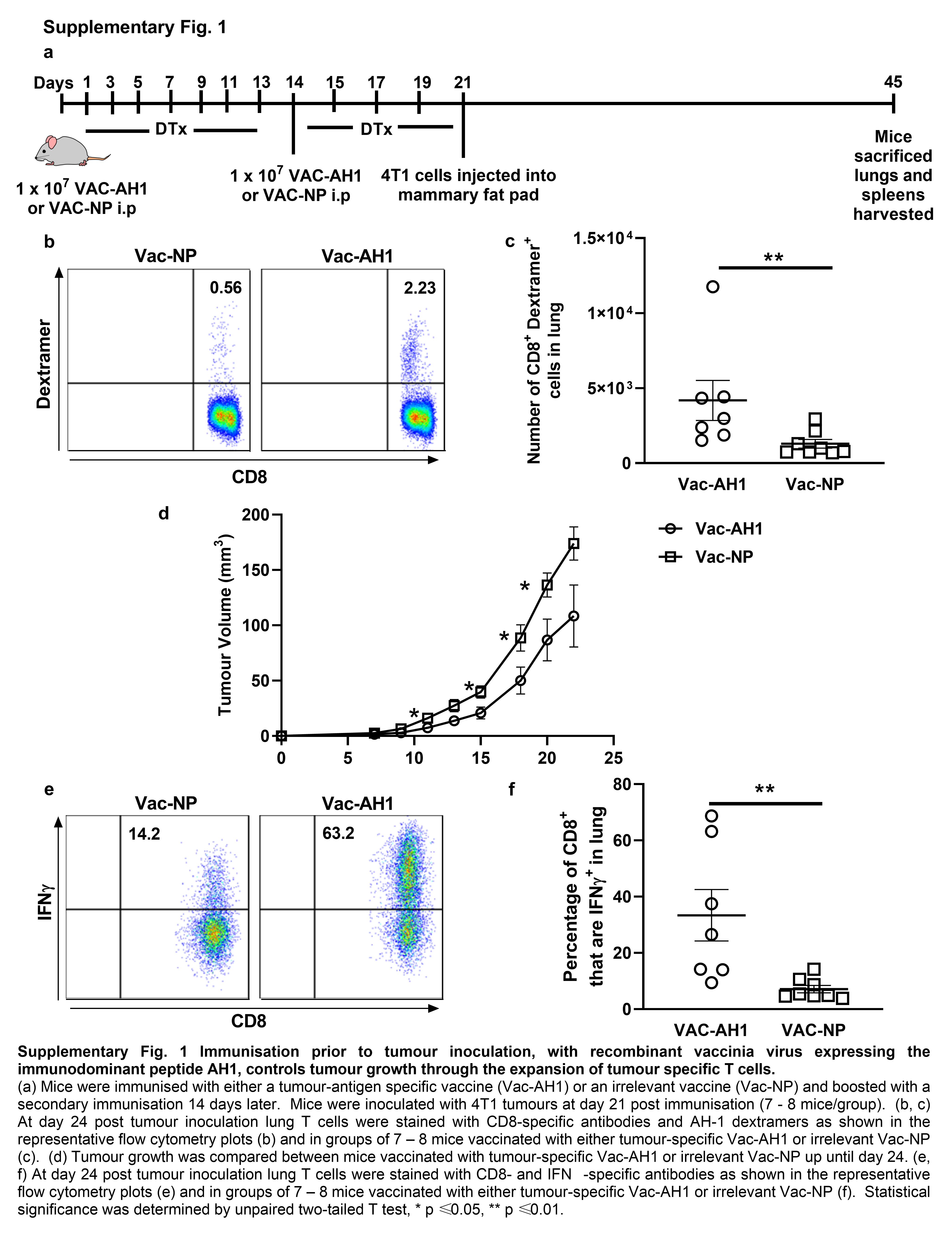

Supplement: Supplementary file 1 — Supplementary file1 (TIF 2810 kb) [file 262_2020_2603_MOESM1_ESM.tif]
